# Critical Path to First-in-Human Batches of ChAdOx Vectors, Including for Emergency Response

**DOI:** 10.3390/vaccines14060509

**Published:** 2026-06-04

**Authors:** Marco Polo Peralta Alvarez, Shawkat Hussain, Andrea Magri, Jacqueline Vieira, Cheelsea Pereira, Faith Vinluan, Matteo N. Barbaglia, Daniel Wright, Susan J. Morris, Emma Bolam, Eleanor Berrie, Teresa Lambe, Tanja Brenner, Richard Tarrant, Sarah C. Gilbert, Catherine M. Green, Alexander D. Douglas

**Affiliations:** 1 Jenner Institute, Nuffield Department of Medicine, University of Oxford, Oxford OX3 7DQ, UK; 2 Clinical Biomanufacturing Facility (CBF), Nuffield Department of Medicine, University of Oxford, Oxford OX3 7JT, UK; 3 Pandemic Sciences Institute (PSI), Nuffield Department of Medicine, University of Oxford, Oxford OX3 7BN, UK; 4 Oxford Vaccine Group, Department of Paediatrics, University of Oxford, Oxford OX3 7LE, UK; 5 CAMS-Oxford Institute, Chinese Academy of Medical Sciences & Peking Union Medical College, University of Oxford, Oxford OX3 7BN, UK

**Keywords:** adenovirus, ChAdOx1, vaccine manufacturing, good manufacturing practice, outbreak response

## Abstract

**Background:** Adenovirus-vectored vaccines played an important role in the global response to SARS-CoV-2. Adenovirus platforms have many advantages including a simple and readily transferred manufacturing process, low cost, and thermostability. Speed of production of an initial Good Manufacturing Practice (GMP)-compliant batch has, however, been viewed as a limitation of adenovirus vectors relative to mRNA platforms. Production of the initial viral starting material and release testing are key rate-limiting steps. **Methods:** Production of viral starting material from DNA, and release testing in accordance with regulatory expectations, for first-in-human trials of adenovirus-vectored vaccines. **Results:** We describe experience of these stages in the production of the first GMP batches for multiple adenovirus-vectored candidates and the adaptations made for ChAdOx1 nCoV-19 (the Oxford COVID-19 vaccine) in early 2020. We also report development of a streamlined approach to starting material generation, enabling initial GMP batch availability within c. 60 days of publication of a new pathogen sequence. Using a New World arenavirus vaccine construct as a proof of concept, we demonstrate reproducible execution of this pipeline, maintaining acceptable infectivity and other quality attributes. **Conclusions:** We discuss opportunities for additional time savings in the future. This work demonstrates suitability of an adenovirus platform to contribute to the “100 Days Mission” for vaccines against “Disease X”.

## 1. Introduction

The speed of vaccine production in response to the COVID-19 pandemic exceeded many expectations, yet the value of further acceleration is clear [1,2].

Adenovirus-vectored vaccine platforms emerged as pivotal during the pandemic, demonstrated by the successful deployment of >3 billion doses of the Oxford/AstraZeneca ChAdOx1 nCov-19 vaccine [2]. Nonreplicating simian adenovirus vectors such as ChAdOx1 are E1/E3 deleted, exhibit low human seroprevalence, and elicit strong cellular and humoral immunity in animals and humans [3,4,5]. They have considerable strengths: simple manufacturing, involving only commonly used bioprocess steps; low cost, with a drug substance cost of goods of <USD 1 per dose; ease of distribution, given their stability at standard refrigerated temperatures (2–8 °C); proven immunogenicity after mucosal administration in humans; and potential for dry formulations such as microneedle patches, enhancing global accessibility [6,7,8,9,10,11].

Slowness of manufacturing is perceived by some observers as a disadvantage of the adenovirus platform, relative to mRNA. The critical path to clinical trial material requires DNA synthesis and cloning, virus rescue by transfection of producer cells, amplification to produce viral starting material (the first stage of viral seed to be used in GMP manufacturing), GMP drug substance (DS) and drug product (DP) manufacture, and release testing [12,13].

We have previously described the scale-up of manufacturing of the ChAdOx1 nCoV-19 drug substance (including the process used at commercial scale), and colleagues have reported issues encountered by a partner in producing viral starting material for another ChAdOx1 candidate [7,12]. We have not, however, described in detail the methods used to manufacture the first clinical batch in early 2020 or other candidates made in-house using the platform over the past decade. Viral starting material production, and to some extent release testing, are the main rate-limiting steps in adenovirus vector production, each currently taking a number of weeks. The step preceding starting material production, namely synthesis of antigen-coding DNA, is not unique to adenovirus: it is shared with other vaccine platforms including mRNA. Once starting material is in hand, adenoviral DS and DP production is relatively rapid (<14 days from inoculation of the production bioreactor). Starting material production and release testing, on the other hand, both involve lengthy biological processes, some of which are unique to the adenovirus platform. Here, we will therefore focus on these stages, as they are the main determinants of the length of the critical path to Phase I trial.

The major requirements of viral starting material production are that it should provide a pure population of the desired virus (free of unwanted product variants or adventitious agents) in sufficient quantity for production of the first GMP batches of DS and master virus seed (MVS), and of suitable quality to meet regulatory expectations. It is not, however, expected in the UK that the starting material will itself be made in accordance with GMP. Our practice has been to manufacture it in accordance with the principles of GMP, guided by a full risk assessment and quality risk management plan. This has involved the use of GMP-appropriate materials and consumables, a fully tested GMP-manufactured bank of producer cells, and a controlled laboratory environment (rather than a graded cleanroom).

It is a regulatory expectation for virus-based vaccines that genetic homogeneity and stability will be demonstrated prior to clinical use. An impure starting material pool containing unwanted product variants may be prone to outgrowth of a variant, and hence genetic instability—a known issue in adenovirus starting material production. Expression of some antigens in the producer cells is known to inhibit adenoviral growth. Variants with mutations in the antigen coding sequence or promoter, abrogating expression of functional antigen, may therefore have a selection advantage and are a particular concern as this would be expected to affect potency. We and colleagues have previously reported instances in which this appears to have occurred, including data suggesting such problems can be reduced but not completely averted by transcriptional repression of antigen expression during vector production [12,14]. An additional concern is the possible presence of the non-viral plasmid sequence (NVPS, e.g., the plasmid origin of replication and antibiotic resistance gene used for adenoviral genome cloning). In contrast, mutations within the vector’s genes of adenoviral origin have never, in our experience, proven problematic (any lacking fitness will not replicate, and replication incompetence in vaccinees’ cells is assured by the E1 deletion, irrespective of changes elsewhere).

Genetic homogeneity of viral starting material is often achieved by plaque purification. We and colleagues previously reported the use of a limiting dilution of a viral lysate, a process termed ‘single virion cloning’ (SVC), but we did not describe our implementation of this in detail [12,14]. SVC has the theoretical advantage over plaque purification of avoiding bias towards a virus with a skewed (florid) CPE phenotype, but both plaque purification and SVC impose a requirement for lengthy and laborious subsequent re-amplification of the virus.

Historically we have determined genetic identity, purity, and stability using a combination of PCR-based assays and Sanger-sequencing, both on the starting material itself and on samples collected after serial passage (to detect instability) [12]. These methods have limitations, notably in detection of minor populations with point mutations, and increasing use of next-generation sequencing (NGS) has been reported in the field.

The deletion of E1 which renders ChAdOx-vectored vaccines replication-incompetent is complemented during manufacturing by the expression of E1 from human adenovirus serotype 5 in trans by HEK293-based producer cells. Acquisition of the deleted E1 gene region from the cells is a special form of genetic alteration which is of particular concern as it could result in replication competent adenovirus (RCA). In the case of human adenovirus serotype 5 products, this can occur by homologous recombination between the E1 flanking regions which are present in both the HEK293 and vector genomes.

After DS and DP manufacture, the assays which are typically rate-limiting for product release are those involving prolonged incubations of cultures. In vitro adventitious viral agent (AVA) and RCA assays are performed on the bulk harvest (sampled before the downstream process [DSP]) and DS, respectively, and last c. 45 days. Sterility is tested for drug product samples: the classical pharmacopoeial assay requires a 14-day incubation.

Here, we describe our experience with the production of starting material to enable Phase I trials using an established process; modifications to this were made for ChAdOx1 nCoV-19 in early 2020. Additionally, we describe a proof-of-concept of an accelerated workflow which we intend to use for future outbreak response.

## 2. Material and Methods

### 2.1. Recombinant DNA Genome Assembly and Rescue

The design and construction of most vaccines mentioned in this report have previously been reported elsewhere; citations are provided where individual vectors are mentioned in the Section 3 (Results). In most cases, antigen-coding sequences were synthesized (Thermo Fisher, Waltham, MA, USA) and cloned in-house into the E1 loci of ChAdOx1 and ChAdOx2 genome-containing bacterial artificial chromosome backbones, as previously described [4]. DNA for use in viral rescue for viral starting material production was then produced by growth of a clone on animal-derived-component-free agar and broth (e.g., Vegitone, Sigma-Aldrich, Burlington, VT, USA) and using column-based purification (e.g., Qiagen EndoFree Plasmid Mega kit, Qiagen, Hilden, Germany).

For ChAdOx1 Junin constructs, the antigen-coding synthetic gene was cloned directly by the synthesis provider into a ChAdOx1 genome-encoding high-copy plasmid backbone (by exchange of the origin of replication from oriS to pBR322).

Adenoviral genomes were released from BACs/plasmids by restriction digestion for ~2 h at 37 °C. Digested insert size was verified by visualization of a sample on a 1% agarose gel. Where stated, to separate the linearized genome from the digestion mixture (including NVPS), preparative low melting point agarose gel electrophoresis was undertaken followed by excision of the linearized genome band and column-based purification (e.g., QiaQuick kit; Qiagen).

In three cases (see below), linear DNA was extracted from a pre-clinical research-grade virus prep by phenol-chloroform extraction.

### 2.2. Transfection, Viral Rescue, and Limiting Dilution

Viral rescue was performed using adherent HEK 293A (from a University of Oxford in-house proprietary GMP Master Cell Bank) or HEK293 TREx (Thermo Scientific, henceforth ‘Trex’), as stated in the Section 3 (Results) for individual products. Adherent cells were maintained at 37 °C and 5% CO_2_ in Dulbecco’s Modified Eagle Medium (DMEM) supplemented with 2 mM L-glutamine and 10% foetal bovine serum from a TSE-free source (all from Thermo Fisher). In the case of Trex cells, medium was additionally supplemented with 5 µg/mL blasticidin (Melford Laboratories, Ipswich, UK).

To the rescue virus, cells were seeded in 6-well plates at 4–8 × 10^5^ cells/well and transfected with linearized DNA 24 h later, at 60–80% confluence. Typically, wells were transfected with complexes of 5 µg linearized vector with 30 µL of Lipofectamine 2000 CD (Thermo Scientific). In three cases (ChAdOx1 Chik; ChAdOx1 MERS; ChAdOx1 Zika), rather than using linearized plasmid DNA, virus was ‘re-rescued’ by transfection into the cells of viral genome phenol-chloroform purified from a pre-clinical virus preparation.

To obtain a genetically homogeneous and stable population of virus, free from NVPS sequences, in most instances prior to 2020 a process of single virion cloning was used. Briefly, rescued virus from the transfection (6–14 days post transfection, when the cytopathic effect (CPE) is evident) was harvested by trituration of cells in the culture medium followed by three freeze–thaw cycles to release the virus, centrifugation at 300× *g* for 10 min, removing cellular debris, and collecting the supernatant, 50% of which was then used to infect a T25 flask at 80–85% confluency. After approximately 3 days, when visual inspection using a light microscope identified significant CPE, the viral material (known as ‘Amp1’) was again harvested using a similar freeze–thaw process and amplified on a T75 flask to generate an ‘Amp2’ preparation by trituration and freeze–thaw in 5 mL of the culture medium. The viral titer of this material was determined using a cell-based infectivity assay (see Section 2.5). SVC was initiated in 96-well plates, each well seeded the day before with 2 × 10^4^ to 4 × 10^4^ viable cells per mL, infected with either one or three infectious units per well. Plates were inspected daily for up to 10 days. When CPE was observed the entire contents of the well (cells and media) was collected by trituration. In some cases (where homogeneous correct clones were not isolated initially) this SVC process was repeated a second time using material amplified from the first round clone.

In most cases since 2020, the SVC process was replaced by a process of limiting dilution of transfected cells (LDTC), rather than virions, typically using adherent Trex cells. Then, 24 h after transfection, a single-cell suspension was prepared by careful trituration of cells in a transfected well (TrypLE Express, Thermo Scientific). A 3-fold dilution series of transfected cells in untransfected cells was prepared and plated in 96-well plates (96wp—60 wells per dilution) pre-seeded with 1.3 × 10^4^ untransfected cells/well. Plates were incubated for 4–8 days and then screened for cytopathic effect (CPE) by light microscopy. Wells containing single foci of CPE were identified, favoring dilutions at which <33% of wells had identifiable CPE. Cells from these wells were harvested and (unless otherwise stated) disrupted by three freeze–thaw cycles, and the resulting lysate was used for further amplification on adherent cells.

In the case of the accelerated amplification workflow, lysis of harvested LDTC wells was performed by addition of 1/5 volume of 5× lysis buffer (250 mM Tris, 5% *v*/*v* polysorbate 20 [PS20], and 25% *w*/*v* sucrose) to the harvested material in a microtube, along with benzonase (Millipore, Burlington, VT, USA) to a final concentration of 15 U/mL, followed by agitation in a Thermomixer (Eppendorf, Hamburg, Germany) at 37 °C and 1400 RPM for 120 min.

### 2.3. Amplification to Generate Viral Starting Material

Viral amplification was performed using HEK 293A or Trex cells (as described above) and maintained in adherent and/or suspension culture (as described in Table 2 for individual products). Adherent culture conditions were as described above.

Suspension-adapted cultures used CD293 medium (Thermo Fisher), with one exception (culture for ChAdOx2 CCHF used BalanCD 293 medium [Fujifilm Biosciences, Santa Ana, CA, USA]), and was performed in Erlenmeyer flasks. In all cases medium was supplemented with 4 mM L-glutamine (both from Thermo Fisher). For Trex cells, 1 µg/mL blasticidin (Melford Laboratories) was also added.

Following limiting dilution selection, historical starting material generation used serial adherent passages in progressively larger vessels, with a switch to suspension culture for the final passages in some cases. At each stage, virus was released from cells by freeze–thaw lysis. When sufficient material had been produced (typically at the third passage), the virus was analyzed by PCR (identity, flank-to-flank, and NVPS), transgene cassette Sanger sequencing, and restriction fragment gel electrophoresis assays as described under Section 2.5 (Analytics) below. Before use, the lysate was subjected either to filtration through 5 µm then 0.45 µm syringe filters, or to virus purification by 2-step caesium chloride density gradient ultracentrifugation as previously described [13]. In one case in which a larger quantity was required by the customer, starting material was purified by tangential flow filtration and anion exchange [6].

For the accelerated workflow, we commenced suspension amplification immediately after the (adherent) limiting dilution step. A total of 100 µL of lysate from a single harvested LDTC well was added to a 30 mL culture of Trex cells in suspension-adapted BalanCD HEK293 medium (Fujifilm Biosciences) supplemented with 4 mM L-alanyl-L-glutamine (Thermo Fisher), agitated at 130 rpm (25 mm orbit) at 37 °C and 5% CO_2_. Given the use of the PS20-containing lysis buffer in the harvest of the LDTC well, the final expected PS20 concentration was 0.003%. Samples were taken daily for viable cell density/viability (determined using Nucleocounter NC202, Chemometec, Allerod, Denmark), and viral genome copy number was determined by digital PCR (dPCR). Cultures were fed every 48 h with ~30 pL/cell BalanCD HEK293 feed (Fujifilm) and diluted to 0.5 × 10^6^ cells/mL once viable cell density exceeded 1.8 × 10^6^ cells/mL. Cells were harvested on the day at which viability fell to <75% by addition to the flask of benzonase to 15 U/mL, incubation for 15 min at 130 rpm and 37 °C, and then addition of 5× lysis buffer (as above) to 1/5 final volume and incubation for a further 2 h at 130 rpm and 37 °C. Lysate was transferred to 50 mL centrifuge tubes and centrifuged at 3000× *g* for 30 min at 4 °C. Supernatant was then aseptically syringe-filtered through 5 µm and 0.45 µm filters, successively, aliquoted, and stored at −80 °C.

### 2.4. Drug Substance Production

Production of DS for the ChAdOx1 nCoV-19 Phase I batch used the single-viral-lifecycle shake-flask-based un-fed batch upstream process in Trex cells grown in CD293 medium supplemented as above. Cells were infected with viral starting material at a multiplicity of infection (MOI) of 2–4 IU/cell and harvested 44–48 h later by centrifugation at 200× *g* for 30 min at 20 °C followed by freeze–thaw lysis in 2.5% culture volume of Cell Lysis Buffer (10 mM Tris, 135 mM NaCl, and 1 mM MgCl) including Benzonase at 500 U/mL for 25–35 min at 18–22 °C after the first thaw. A bulk harvest lot was prepared by clarification of this material by centrifugation at 1911× *g* for 30 min at 4 °C and stored at −70 to −85 °C. DS was then purified from this by 2-step caesium chloride density gradient ultracentrifugation as previously described [13]. Buffer exchange was performed by 3× dialysis into formulation buffer A438, followed by dilution and filtration with a 0.45 µm filter.

To exemplify the accelerated workflow for ChAdOx1 Junin, we used a two-viral-lifecycle fed batch upstream process similar to that used for commercial-scale production of ChAdOx1 nCoV-19. Suspension Trex cells in BalanCD HEK293 medium with 4 mM Glutamax/L-alanyl-L-glutamine (Thermo Fisher) in a single-use stirred tank bioreactor were infected at a cell density of 0.8 × 10^6^ cells/mL and MOI of 0.1 IU/cell and harvested 120 h later, as previously described [7]. In this instance, BioBlu 10 c vessels with macrospargers and BioFlo320 controller (both from Eppendorf) were used.

### 2.5. Analytics

PCR assays for transgene identity, transgene length/purity (flank-to-flank), and absence of NVPS were conducted as previously described [12]. Methods for viral particle (VP) concentration determination by UV spectrophotometry (A260) and infectious unit (IU) titration by hexon immunostaining were also conducted as previously reported [5,15]. Briefly, the infectivity assay used HEK293A cells, and immunohistochemical detection of hexon used a mouse anti-hexon antibody (Abcam, Cambridge, UK).

Mycoplasma NAT assays were performed by SGS Vitrology using the Applied Biosystems MycoSEQ™ Real Time Polymerase Chain Reaction (PCR) Kit (Thermo Fisher). Endotoxin levels were determined using the Endosafe^®^ nexgen-PTS™ limulus amoebocyte lysate (LAL) test kit (Charles River Laboratories, Wilmington, DE, USA).

A non-pharmacopoeial ‘sterility check’ assay was performed by inoculation of Thioglycollate Broth and Trypticase Soy Broth (TSB) (Biomerieux, Marcy l’Etoile, France) with starting material to check for lack of growth within at least 14 days at 22.5 ± 2.5 °C (TSB) and 32.5 ± 2.5 °C (both media), before viral starting material was transferred to the cleanroom.

Assays of genetic stability (typically via identity and flank-to-flank PCR as above, plus transgene cassette Sanger sequencing and restriction fragment length analysis of extracted genomes) were performed after 5–10 serial passages on adherent cells.

Assays for detection of adventitious organisms in the ChAdOx1 nCoV-19 GMP bulk harvest lot comprised pharmacopoeial assays of bioburden and endotoxin, absence of mycoplasma spp by qPCR, absence of retroviral contamination using the fluorescent probe-based product-enhanced reverse transcriptase assay (FPERT), and a panel of validated PCR assays targeting human pathogens. The PCR panel included the following: adeno -associated Virus; Human Herpes Viruses (including Human Cytomegalovirus, Epstein Barr Virus, Human Herpes Simplex Virus types 1 and 2, and Human Herpes Viruses (6, 7, and 8), and Varicella-zoster Virus; Human Enteroviruses; Human Rhinovirus; Rubella Virus; Mumps Virus; Measles Virus; Human Parainfluenza Virus (types 1, 2, and 3); Human Respiratory Syncytial Virus (A and B); Reoviruses (types 1, 2, and 3); Human Coronaviruses and Influenza Viruses. With the exception of bioburden and endotoxin, these assays used proprietary methods and were performed by SGS Vitrology, Glasgow, with a turn-around time <7 days.

Assays of encapsidated viral genome copy titers (GC/mL) during single-step suspension amplification and the ChAdOx1 Junin DS upstream process were performed using a digital PCR (dPCR) assay. Samples were processed as previously described for qPCR [6]. The forward and reverse primers were 5′-GTGGGAAAAGTGACGTCAAACGAG-3′ and 5′-TGCATCCGCCTAGAAACACCTCA-3′, respectively. A probe with sequence 5′-GAGAGCGCGGGAAAATTGAGTATT-3′ was labelled with 6-carboxyfluorescein (6-FAM) and Black Hole Quencher. Assays were run on a QIAcuity Four instrument using 96-well 8.5k partition QIAcuity nanoplates (both from Qiagen). To provide conservative estimates of titers (given that re-annealing of single DNA strands after the heat treatment in this processing workflow is known to be incomplete), raw results were divided by two to infer double-stranded DNA genome concentration.

Particle: infectivity ratios (P:I) were calculated from infectivity titers and physical titers (based upon A260 for CsCl-purified material or dPCR for impure material).

For viral whole-genome sequencing, viral DNA was extracted using a QIAamp DNA Blood Mini Kit (Qiagen). A library was prepared using a NEBNext UltraII FS DNA Library Prep Kit for Illumina (NEB, Ipswich, MA, USA), and it was sequenced on an iSeq100 instrument (Illumina, San Diego, CA, USA), all in accordance with the manufacturers’ instructions. Sequence information was analyzed using Geneious (versions R11.1 to 2025.0, Dotmatics, Boston, MA, USA).

### 2.6. Data Analysis

Graphs were prepared using Prism 10.0 (Dotmatics, Boston, MA, USA).

## 3. Results

### 3.1. Pre-Pandemic Viral Starting Material Production

**Figure 1:**
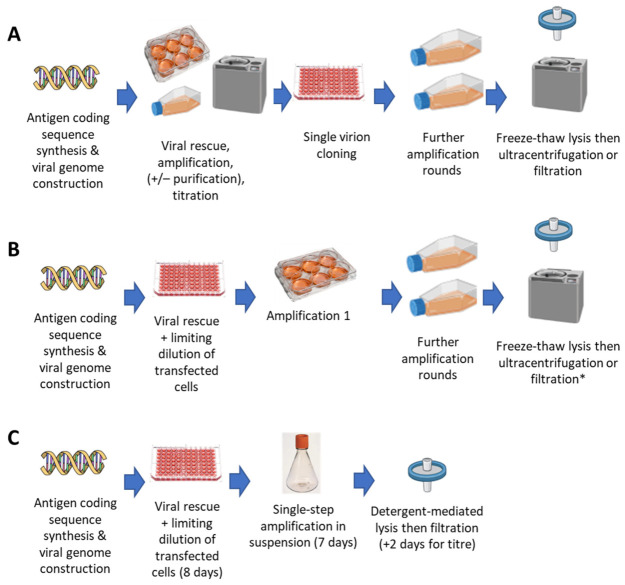
Workflows for starting material production. (**A**) Pre-pandemic workflow incorporating single virion cloning (SVC), typically taking 8–16 weeks from the point of transfection for viral rescue (see Table 2 regarding durations for individual campaigns). (**B**) Workflow used for ChAdOx1 nCoV-19 and other post-pandemic candidates, incorporating limiting dilution of transfected cells (LDTC), typically taking 6–10 weeks from transfection to starting material. * Indicates that in one case, TFF and anion exchange were used, as per Table 2. (**C**) Accelerated workflow exemplified in this manuscript for ChAdOx1 Junin, with single-step amplification in suspension, including step durations, resulting in a 17-day timeline from transfection to availability of starting material. Figure created using BioRender (https://www.biorender.com, accessed 20 January 2026).

Our pre-pandemic viral starting material production pipeline and testing package are summarized in Figure 1 and Table 1, respectively.

In campaigns initiated between 2015 and early 2020, starting materials were successfully manufactured for eleven different ChAdOx1/2-vectored candidates. Results obtained across these campaigns are summarized in Table 2.

Among these pre-pandemic products, gel purification of the linearized genome was performed for four of the eight vectors for which production began with rescue from linearized plasmid DNA (rather than DNA extracted from a pre-clinical virus preparation). This did not appear necessary or effective for the removal of NVPS: NVPS-free virus could be obtained by SVC after rescue by transfection of the unpurified linearization digestion product, and some NVPS-containing isolates were obtained even when gel purification was performed.

Ten underwent at least one round of SVC, followed by three to six stages of amplification (of which zero to two were performed in suspension). One, made in 2016 (ChAdOx1 MenB.1), was made directly from ‘polyclonal’ rescue by transfection of a whole vessel of cells: at that point we viewed SVC as a ‘troubleshooting’ method applicable to vectors known to be susceptible to genetic instability and unnecessary for others, whereas we subsequently adopted it as routine.

Nine starting materials were purified by CsCl gradient ultracentrifugation. From 2019 onward this was not always performed: filtered culture lysate was sometimes used as the starting material, with satisfactory results. In other cases customers continued to prefer preparation of purified starting material, due to the extended analytical characterization this permits and its somewhat greater track record of stability in long-term storage.

In each of these eleven cases, the starting material conformed to specification (see Table 1, including genetic identity, purity, and stability) as did all additional test articles from subsequent DS/DP production (including freedom from adventitious agents and RCA).

Four starting material production campaigns were unsuccessful, in each case due to mutations arising in the antigen expression cassette. From 2019 onwards, we began using Trex cells during starting material production. When used in combination with an appropriately modified promoter in the vector’s antigen expression cassette, these cells repress the expression of the vaccine antigen during production. We have previously described the beneficial effect this can have upon genetic stability and productivity [6,14]. Since we began routine use of antigen repression and avoidance of runs of >5 consecutive identical nucleotides, we have not observed instability of the antigen sequence in any starting material which has been prepared for GMP manufacturing. Genetic instability believed to be related to genome size has continued to be observed in some pre-clinical preparations with large transgene inserts but is outside the scope of the current manuscript.

Pre-pandemic starting material preparation campaign durations were typically 8 to 16 weeks (from the availability of the genome-encoding plasmid to the freezing of the purified starting material but excluding synthesis of the antigen-coding DNA and cloning and release testing). Major contributors to this were the rescue and SVC process (c. 4 weeks) and multi-stage amplification (c. 1 week per step—although this could in principle be compressed by weekend work).

GMP production for six of these eleven products involved production of a GMP master viral seed stock (MVS) which was then used to infect the GMP DS production batch. For the remaining five, we proceeded from the starting material to produce a GMP master viral seed stock (MVS) in parallel with the GMP DS batch, rather than sequentially (i.e., the GMP DS batch was derived from cells infected directly with the starting material, not the MVS). Multiple Clinical Trial Authorisation applications including this approach have been accepted by the Medicines and Healthcare products Regulatory Agency.

### 3.2. Starting Material Production and Release Testing for Phase I Batch of ChAdOx1 nCoV-19

In February–March 2020, we sought to shorten timelines for the production of the first GMP batch of ChAdOx1 nCoV-19 (the candidate which subsequently became Vaxzevria, the Oxford/AstraZeneca COVID-19 vaccine).

For some stages of the process, there were obvious options for acceleration without scientifically meaningful change to our usual practices. Several days could be saved from the amplification stage of starting material production by weekend working. In addition, we decided to transfer viral starting material into the cleanroom for DS production in advance of the availability of full assay results (Table 1). We reasoned that unavailability of certain results at that point (for example sterility and mycoplasma) posed a business risk but not a risk to participant safety or scientific validity of trial data, as full results would be available before release of the vaccine for trial use (and clearly material would not be used clinically if any test failed).

To enable more rapid starting material generation while maintaining homogeneity, we developed an alternative method to SVC that we term limiting dilution of transfected cells (LDTC). This avoids the need to amplify and titer the virus after the initial rescue in order to perform SVC at a known MOI. Instead, LDTC relies on the logic that viral rescue is a rare event among a population of transfected cells. Virus originating from a single transfected cell cannot be guaranteed to originate from rescue from a single genome backbone molecule, but this is the most likely outcome. It will certainly be more homogeneous than viruses from a bulk population of transfected cells, and adequate homogeneity can be analytically verified without requiring knowledge of its molecular origin. Together with the acceleration of amplification by weekend working, replacement of SVC by LDTC enabled production of 9.2 × 10^8^ IU of starting material from the adenoviral genome-coding plasmid within six weeks.

We anticipated the need for further changes regarding other aspects of the production and testing process. We therefore initiated quality risk assessments and, guided by these, initiated a dialogue with the UK Medicines and Healthcare Products Regulatory Agency (MHRA) Coronavirus Incident Group regarding regulatory acceptability.

To accelerate DS production, immediate cleanroom availability was required to initiate production cell expansion in parallel with viral starting material production. We therefore paused the campaign which had been occupying our suite in February 2020 (another adenovirus-vectored vaccine) and initiated cell expansion without an intervening fumigation of the suite. We provided evidence for absence of the previous product by PCR-based testing, and our prior data from previous campaigns supported the effectiveness of our cleaning regime.

With respect to adventitious viral agent testing, we considered risks of animal-derived viruses to be adequately mitigated by the use of animal-origin free materials throughout the starting material and DS/DP processes (with the exception of traceable irradiated TSE-free serum), by previous testing of the master cell bank for a full panel of adventitious agents, and by environmental controls. We therefore considered the major risk to be operator-derived viruses. We proposed that, for adventitious virus testing of the DS bulk harvest lot, we could rely upon a PCR panel targeting potential operator-derived viruses (see Section 2), with omission of culture-based in vitro or in vivo virus detection. This approach was consistent with WHO guidance on Ebola vaccine quality, which was considered a relevant precedent. This guidance states ‘it could be discussed (and should be agreed with the National Regulatory Authority) whether to evaluate the possibility of delaying in vitro testing for extraneous agents (viral pathogens and mycoplasmas) at the cell harvest or bulk substance stages or replacing it with validated PCR tests.’ The PCR panel was negative for all tested agents.

We considered the risk of participant harm due to RCA to be negligible for multiple reasons: the 5′ and 3′ E1 flanks in ChAdOx1 have only 42% and 58% homology, respectively, with the human adenovirus serotype 5-derived flank sequences in HEK293 cells, strongly reducing probability of recombination [31]; no RCA had ever been detected in any of 12 GMP ChAdOx batches tested using the conventional assay, nor come to our attention in any of hundreds of pre-clinical ChAdOx preparations; even if E1 were restored, the E3 deletion would provide some residual attenuation; the minimal number of passages between LDTC and harvest would minimize the opportunity for RCA formation; and trial volunteers would be screened carefully for immunocompromise. A negative RCA result was not therefore deemed necessary for initial batch release (testing was, however, initiated, and the result was subsequently confirmed to be negative).

Bulk harvest was shipped for testing on 25 March 2020, and DP was filled on 2 April 2020. The last result required to release the batch for trial was the sterility test (performed by Bioreliance), received on 20 April 2020, i.e., 18 days after completion of DP manufacture.

### 3.3. Post-Pandemic Acceleration of Starting Material Production

Since adopting LDTC for ChAdOx1 nCoV-19, we have used this method to produce eight further ChAdOx1 and ChAdOx2 starting materials, all using antigen-repressing cells (Table 2). Six of these campaigns used four or five adherent amplification stages, taking five to seven weeks from plasmid to purified starting material. Two used three adherent amplifications plus additional suspension stages to generate higher yields of material, one of which required multiple additional amplifications and a chromatographic DSP to generate an adequate yield, taking ten weeks. All conformed to specification, as did all additional test articles from subsequent DS/DP production for the five that have gone on to subsequent GMP production, including freedom from adventitious agents and RCA. Since 2020, no ChAdOx starting material production campaign has been unsuccessful (consistent with the improved results since adoption of antigen-repressing cells, as described in Section 3.1).

This experience demonstrates acceleration from our pre-pandemic SVC-based methods, but we wished to further accelerate the method for suitability for future emergency response.

We initially accelerated plasmid production by arranging for a gene synthesis provider to directly clone the newly synthesized antigen-coding sequence into an adenoviral backbone plasmid and supply this at a suitable quality to be linearized and used directly in the rescue of starting material to be used for GMP. This saves at least 3 days of cloning and DNA preparation and was enabled by insertion of a high-copy origin of replication (pBR322) in the genome-backbone-containing plasmid. Sequencing of the constructs made using the high-copy plasmid has not revealed any change in rates of mutation as compared to our previous bacterial artificial chromosome-based method.

We then sought to accelerate the starting material amplification stage. We had previously suggested that a single adherent amplification and a single suspension amplification may be sufficient to produce adequate starting material for production of trial material using a two-viral-lifecycle ‘low MOI’ process and provided preliminary data as a proof of concept [1]. We sought here to achieve further acceleration by completely eliminating the adherent amplification stage (transferring virus from harvest of a single well on a 96wp to amplification in suspension in a shake flask).

**Figure 2:**
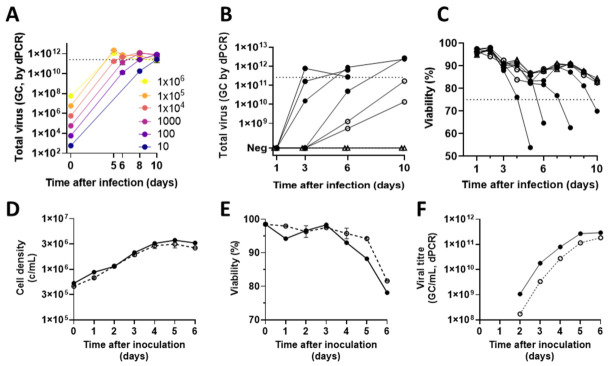
Amplification in suspension-adapted HEK293 TRex cells results in suitable starting material for DS production for a Phase I trial. (**A**) Continuous viral amplification in suspension is robust to amount of input virus. Varying numbers of infectious units of virus (as indicated by the colors annotated in the legend) were added to Trex cells in Erlenmeyer flasks, maintained as described for accelerated amplification in suspension, with 0.003% PS20, and viral titer was determined by dPCR. Points indicate mean and error bars (where visible) for the range of two flasks within one experiment; data shown is representative of duplicate experiments. Results are expressed as total virus to allow comparison given varying flask volumes after varying dilutions. Day 0 values indicate the inputs in terms of GC (as opposed to IU). Dotted line indicates 2.6 × 10^11^ GC, the quantity required for infection of an 8 L culture at 8 × 10^5^ cells/mL with 0.1 IU/cell, assuming P:I 100 and allowing a 4-fold safety factor, e.g., for losses on harvest. (**B**,**C**) Viral growth (**B**) and cell viability (**C**) after transfer of LDTC wells to suspension amplification in each of eight flasks. Filled circles indicate four flasks in which productivity reached the threshold of 2.6 × 10^11^ GC, as described above, by day 10; open circles indicate two flasks in which amplification was detectable but did not reach this level by day 10; open triangles indicate two flasks in which no viral growth was detectable. Data is representative of two similar experiments. (**D**–**F**) Cell count (**D**), viability (**E**), and viral titre kinetics (**F**) after infection of a bioreactor with starting material produced using the above accelerated workflow (filled symbols and solid line, *n* = 1), with comparator data from bioreactors using conventionally produced starting material (open symbols and dashed line, *n* = 2, and points indicate median and error bars indicate range). In each case the vessel was inoculated with cells on day 0 and infected with viral starting material at an MOI of 0.1 IU/cell on day 1.

We anticipated that output from LDTC wells would be low and variable, and hence amplification would take several days, with some variability. We therefore planned a regimen of cell dilution and feeding to maintain cell health during an uncertain period of growth. We initially assessed how this performed with a wide range of input quantities of previously titered virus. We found that virus amplification was robust to input virus quantities across five orders of magnitude (Figure 2A). Although we were concerned that use of detergent-containing lysis buffer in LDTC harvest might impact subsequent amplification, presence of a final concentration of up to (but not more than) 0.003% PS20 in the flask did not affect cell growth. The harvest trigger of viability <75% was reached 5–10 days after infection. Allowing for losses on harvest, sufficient virus was produced to infect a 10 L bioreactor (calculation described in caption for Figure 2).

We had previously suggested purification of starting material from a shake flask lysate by syringe-driven depth filtration and anion exchange, resulting in removal of PS20 before the addition of the starting material to the production bioreactor [1]. To minimize risk of contamination or operator error, we explored the simpler alternative of centrifugal clarification of the shake flask lysate followed by 0.45 µm filtration, relying on a dilution factor of ≥333-fold to achieve an in-bioreactor PS20 concentration ≤ 0.003%. Losses using this method were ≤75%.

We then proceeded to test this approach using output from true LDTC wells, in the context of a workflow spanning transfection, LDTC, and amplification. Having observed that viral release by freeze–thaw lysis can sometimes be incomplete, we harvested the 96-well LDTC plates with PS20 detergent-mediated lysis. We observed satisfactory amplification across three experiments, with each experiment providing at least two candidate starting materials of suitable titer (Figure 2B,C and Table 3). For each experiment, LDTC wells were harvested eight days after transfection, and at least one flask was harvested with satisfactory productivity within seven days of the beginning of amplification, indicating a total of 15 days from transfection to availability of starting material with a GC/mL titer or c. 17 days with IU/mL titer.

The use of this method results in the presence of an appreciable quantity of detergent in the viral starting material and hence in the DS production culture (PS20 at ≤0.003%). Although we had observed satisfactory amplification in the presence of 0.003% PS20 in shake flasks, we noted a previous report of an unexpectedly detrimental effect of another detergent (PS80) upon adenovirus production in bioreactors, unpredicted by experience with unsparged cells [32]. We therefore tested the suitability of viral starting material produced using this workflow in a 10 L bioreactor, using the previously described ‘low MOI’ fed batch method [7]. Results were similar to our previous experience using other viral seeds in the same process, both in terms of cell growth and in terms of viral productivity (Figure 2D–F). Productivity at harvest was 2.9 × 10^11^ GC/mL and 2.1 × 10^9^ IU/mL in a volume of 8.9 L, indicating a satisfactory P:I ratio of 138. Whole-genome sequencing indicated satisfactory homogeneity of the virus, with a mean read depth of 3340 and <2% mismatch from the reference genome at any locus.

This would provide sufficient DS both for production of c. 10,000 doses of drug product and for sufficient MVS for >10,000 L of future DS/WVS production. These calculations assume DSP recovery of 50% (typical in our previous data [7]), a requirement for 8 × 10^7^ IU of MVS to infect 1 L of cells at 8 × 10^5^ cells/mL and MOI of 0.1 IU/cell, and doses of 5 × 10^10^ VP of DP, with 50% loss of DS during filling and QC.

## 4. Discussion

Acceleration of the manufacture of the first clinical batch of a novel vaccine against an emerging pathogen could have considerable impact in a future emergency. In the case of adenovirus-vectored vaccines, the basic principles of vaccine design and production are well established, but the critical path to a first GMP batch has not previously been described in detail for the ChAdOx simian adenovirus vector platform (or, possibly, for other adenovirus vectors). Here, we have reported results obtained in producing a total of 20 ChAdOx vaccine candidates for Phase I clinical trials and subsequent changes to improve suitability for emergency response.

Iterative improvement over the course of multiple batches over a number of years had resulted in reasonably robust—but not particularly rapid—processes for ChAdOx starting material by the time of the COVID-19 outbreak in 2020. Under the emergency circumstances of the pandemic, considerable time savings were made both in starting material production and in release testing (respectively, by LDTC and by release based upon molecular rather than culture-based assays). Nonetheless, there was a large element of good fortune in our ability to manufacture and release the Phase I batch as quickly as we were able to. The effort was critically vulnerable to the loss of any of a handful of individual experienced staff members, and it was simply luck that the facility had been manufacturing a similar product (and hence was stocked with all necessary materials and consumables) at the time of the outbreak. Moreover, the commitment of MHRA’s resources to provide rapid consideration and responses to our proposals was critical.

Combining LDTC with single-step amplification in suspension, as described here, can accelerate starting material production to c. 15 days from transfection to harvest. This suggests feasibility of release of a Phase I trial batch (and MVS for future DS/working virus seed production) in <60 days from publication of a novel pathogen sequence (allowing 14 days for DNA synthesis, cloning and shipping, 7 days for starting material infectivity and bioburden testing, 7 days from infection of the DS production bioreactor to DP filling, and 17 days for release testing, shipping, and certification). Importantly, this simplified pipeline is less labor-intensive and therefore cheaper and easier to implement at the earliest stages of an outbreak, when it is generally unclear whether a true emergency will arise. Avoidance of lost time in outbreak response requires that the early steps can be executed with modest resources (as were available in early January 2020, not late March). This simplification of starting material production shifts the key resource allocation decision to the point at which production cell thaw and expansion must begin in a GMP suite. To enable the c. 60 day sequence-to-release timeline, our current DS process would require a funding commitment for GMP cell expansion to begin within c. 12 days of pathogen sequence publication.

We believe there remains considerable scope for further improvement of these processes. Application of improved modern sequencing and multiplex PCR assays, qualified for adenovirus samples, would simplify and strengthen both the characterization of the product (including any heterogeneity or RCA) and detection of adventitious agents. We believe this is likely to be needed to accelerate release of Phase I batches outside the context of a clear national emergency recognized by the national regulatory authority. A more radical approach would be to entirely bypass the manufacture of viral starting material, including the SVC/LDTC ‘bottleneck’, and instead produce early-phase GMP batches of adenovirus by bulk transfection of producer cells with plasmid DNA in a cleanroom, followed by limited amplification. This would represent a further move away from the hierarchical viral seed bank strategy classically used for virus-based vaccines and would instead have similarities to the manufacture of gene therapy vectors. It would require regulatory acceptance that any resulting vector heterogeneity (including possible presence of NVPS) was well characterized and would not result in risk to trial participants.

Streamlining of both starting material production and release testing are also of clear relevance outside the context of emergency response. The costs of small-scale adenovirus drug substance and drug product manufacturing can be low. In our experience, starting material production and release testing are major contributors to the cost of production of *any* new adenovirus vector to GMP, and hence to barriers hindering clinical evaluation of a wider range of candidate adenovirus-vectored vaccines.

## 5. Conclusions

Production of the first GMP batch of a novel adenovirus-vectored vaccine could be accelerated for future outbreak response, relative to the speed of ChAdOx1 nCoV-19 production in 2020, without compromising product quality or safety.

## Figures and Tables

**Table 1:** Pre-pandemic starting material testing package and specification. Genetic stability testing was also performed by serial passage (5–10 passages beyond the starting material), typically followed by PCR (identity and transgene cassette flank-to-flank), transgene cassette Sanger sequencing, and enzymatic restriction analysis of viral DNA. In some cases additional for-information-only testing was performed using unqualified assays, including next-generation sequencing of viral genomes.

Attribute	Assay	Specification
Virus particle concentration (if purified)	UV spectrophotometry (A260)	Report result
Infectivity	Hexon immunostaining	Report result
Virus-particle-to-infectious-virus-titer ratio	Calculation	Report result
Mycoplasma	qPCR	Negative
Endotoxin	LAL assay	<5.0 EU/mL
Sterility Check	Inoculation	No Growth
Transgene identity	Identity PCR	Positive for insert
Transgene length and purity	Flank-to-flank PCR	Correct sizes
Non-viral plasmid sequence	NVPS PCR	None detected
Antigen expression cassette sequence	Sanger sequencing	Matches reference
Genome integrity	Enzyme restriction analysis (ERA)	Matches expected pattern

**Table 2:** Successful ChAdOx1 and ChAdOx2 starting material production campaigns at University of Oxford Clinical Biomanufacturing Facility, 2015–2025.

Construct (with Citation, Where Available)	Project Start (Year)	Expression Cassette	NVPS Removal by Gel Excision	Antigen Repression	SVC (Figure 1A) or LDTC (Figure 1B)	Amplification Rounds in Adherent Cells	Amplification Rounds in Suspension	Starting Material Purification	Duration (Weeks)
ChAdOx2 HAV [16]	2015	Bacterial antigen string	Yes	No	SVC	4	0	CsCl	9
ChAdOx1 h5T4 [17]	2015	Cancer self-antigen	No	No	SVC	4	0	CsCl	9
ChAdOx1 MERS [18]	2016	Viral glycoprotein	n/a	Yes	SVC	3	0	CsCl	8
ChAdOx1 MenB.1 [19]	2016	Bacterial antigen	Yes	No	Neither	3	0	CsCl	5
ChAdOx1 LS2 [20]	2016	Parasite dual antigens	No	No	SVC	3	0	CsCl	8
ChAdOx1 Chik [21]	2017	Viral glycoprotein	n/a	No	SVC	3	0	CsCl	8
ChAdOx2 RabG [22]	2018	Viral glycoprotein	Yes	No	SVC (2 rounds)	5	1	CsCl	16
ChAdOx1 Plague [23]	2018	Bacterial protein	Yes	No	SVC (2 rounds)	3	0	CsCl	13
ChAdOx1 Zika [24]	2018	Viral glycoprotein	n/a	Yes	SVC	4	0	CsCl	9
ChAdOx1 biEBOV [25]	2019	Two viral glycoproteins	No	Yes	SVC (2 rounds)	5	0	No	13
ChAdOx2 CCHF [26]	2020	Viral glycoprotein	No	Yes	SVC (2 polyclonal amps)	3	2	No	11
ChAdOx1 nCov-19 [27]	2020	Viral glycoprotein	No	Yes	LDTC	4	0	No	6
ChAdOx1 NipahB [28]	2020	Viral glycoprotein	Yes	Yes	LDTC	4	0	CsCl	7
ChAdOx1 LassaJ [29]	2020	Viral glycoprotein	Yes	Yes	LDTC	5	0	CsCl	10
ChAdOx1 E *	2021	Viral glycoprotein	No	Yes	LDTC	4	0	No	6
ChAdOx1 E6 *	2021	Viral glycoprotein	No	Yes	LDTC	4	0	No	6
ChAdOx1 Marburg [30]	2022	Viral glycoprotein	No	Yes	LDTC	4	0	No	6
ChAdOx1 X *	2023	Viral glycoprotein	No	Yes	LDTC	4	0	No	6
ChAdOx1 H *	2024	Antigen string	No	Yes	LDTC	3	1	CsCl	7
ChAdOx2 Y *	2024	Synthetic cancer antigen	No	Yes	LDTC	3	3	TFF-AEX-TFF	10

Stated duration is from transfection to purification (if performed). Some campaigns were discontinuous: for these, duration is estimated ‘active’ time, assuming one week for DNA prep, four weeks for SVC (transfection/amplification/purification/titration/SVC), one week for LDTC, then one week for each round of amplification and one additional week for each purification step. ChAdOx1 nCoV-19 was made in a continuous campaign including weekend working; in this case stated duration is actual calendar weeks (later, a second starting material batch was made to ensure sufficient supply for pandemic manufacture). These timelines include in-process testing, where results are required to progress starting material production, but not testing of the starting material itself. Certain campaigns used individualized approaches. NVPS removal was not applicable for ChAdOx1 Chik and ChAdOx1 MERS because the DNA used was extracted from viruses, rather than linearized from BAC. ChAdOx1 MenB.1 used neither SVC nor LDTC, as discussed in Section 3.1. For ChAdOx2 Y, an unusually large quantity of starting material was required, and so this campaign used our previously described scalable purification method, comprising tangential flow filtration (TFF) and anion exchange (AEX) [6]. NVPS—non-viral plasmid sequence; SVC—single virion cloning; LDTC—limiting dilution of transfected cells. Asterisks indicate anonymized unpublished constructs.

**Table 3:** Outcome of LDTC product amplification in suspension followed by simplified harvest. Rows indicate independent experiments. Each column shows the number fulfilling criterion out of the number evaluated as well as the percentage. Column A indicates the number of flasks in which viability fell to <75% on or before day 10 (out of the total number of flasks inoculated with lysate from an LDTC well). Column B indicates the number of flasks in which total pre-harvest productivity appeared adequate at the time viability fell beneath 75% (out of the total flasks reaching viability <75% and based upon the 2.6 × 10^11^ GC criterion described in the legend for Figure 2). Column C indicates the number of flasks for which post-harvest genome titers were >4 × 10^9^ GC/mL (adequate for infection of a 10 L bioreactor on the basis of an MOI of 15 GC/cell and >333-fold dilution of the starting material to reduce PS20 to <0.003%). Similarly, Column D indicates the number of flasks for which post-harvest infectivity titers were >2.7 × 10^7^ IU/mL (adequate for infection of a 10 L bioreactor at 0.1 IU/cell). For runs 1–2, only a subset of flasks fulfilling the viability criterion were actually harvested, and for each experiment, only two candidate flasks were subjected to infectivity titration. To verify the sequence of the resulting virus, NGS was performed on the DS produced using material from run 1 with satisfactory results (see main text) and additionally on the six candidate starting materials produced in run 3 (3/6 were satisfactory).

Exp	A: Flasks Reaching Viability <75%	B: Adequate Pre-Harvest GC Productivity	C: Adequate Post-Harvest GC/mL Titer	D: Adequate Post-Harvest IU/mL Titer
1	4/8 (50%)	4/4 (100%)	2/3 (67%)	2/2 (100%)
2	3/9 (33%)	Not tested	2/2 (100%)	2/2 (100%)
3	6/6 (100%)	Not tested	Not tested	6/6 (100%)

## Data Availability

The data included in this study are available upon reasonable request.
